# Dopamine release after acute sleep deprivation: culprit of affective state transitions

**DOI:** 10.1002/mco2.630

**Published:** 2024-07-01

**Authors:** Ju Lan, Zhong Chen, Heming Cheng

**Affiliations:** ^1^ Key Laboratory of Neuropharmacology and Translational Medicine of Zhejiang Province, Department of Neurology, The First Affiliated Hospital of Zhejiang Chinese Medical University (Zhejiang Provincial Hospital of Chinese Medicine), School of Pharmaceutical Sciences Zhejiang Chinese Medical University Hangzhou China

1

Affective disorder is a prevalent chronic mental illness characterized by episodes of mania, depression, or alternating recurrent episodes of both. It is associated with a high suicide rate and a low diagnosis rate, leading to severe functional impairments. The disruption of sleep rhythm is a significant contributing factor in affective state transitions, but the underlying cellular and neural circuit mechanisms remain poorly understood. In a recent publication, Wu et al. developed an innovative mouse model of automatic sleep deprivation, unveiling that acute sleep deprivation (SD) leads to abnormal excitability in distinct dopaminergic (DAergic) subsystems, thereby triggering a transition to a manic‐like state and reversal of a depression‐like state in mice.^1^

Firstly, Wu et al. established an automatic SD mouse model by integrating an elevated platform and a rotating beam, effectively reducing both non‐rapid eye movement sleep and rapid eye movement sleep in mice while minimizing stress and compulsive motion (Figure 1A).^1^ They found that normal mice exhibited heightened levels of hyperactivity, aggression, and sexual behavior following 12 h of acute SD. However, this phenomenon reverted to a basal state within 24–48 h, which is highly likely associated with the restoration of sleep‐wake rhythm and warrants further verification. In addition, acute SD significantly ameliorated depression‐like behaviors induced by learned helplessness in mice. Interestingly, this antidepressant effect was sustained for at least 72 h, aligned with the clinical test.^2^ These findings indicate that acute SD can induce a hyperactive state transformation and reverse a depression‐like state in mice.

**FIGURE 1:**
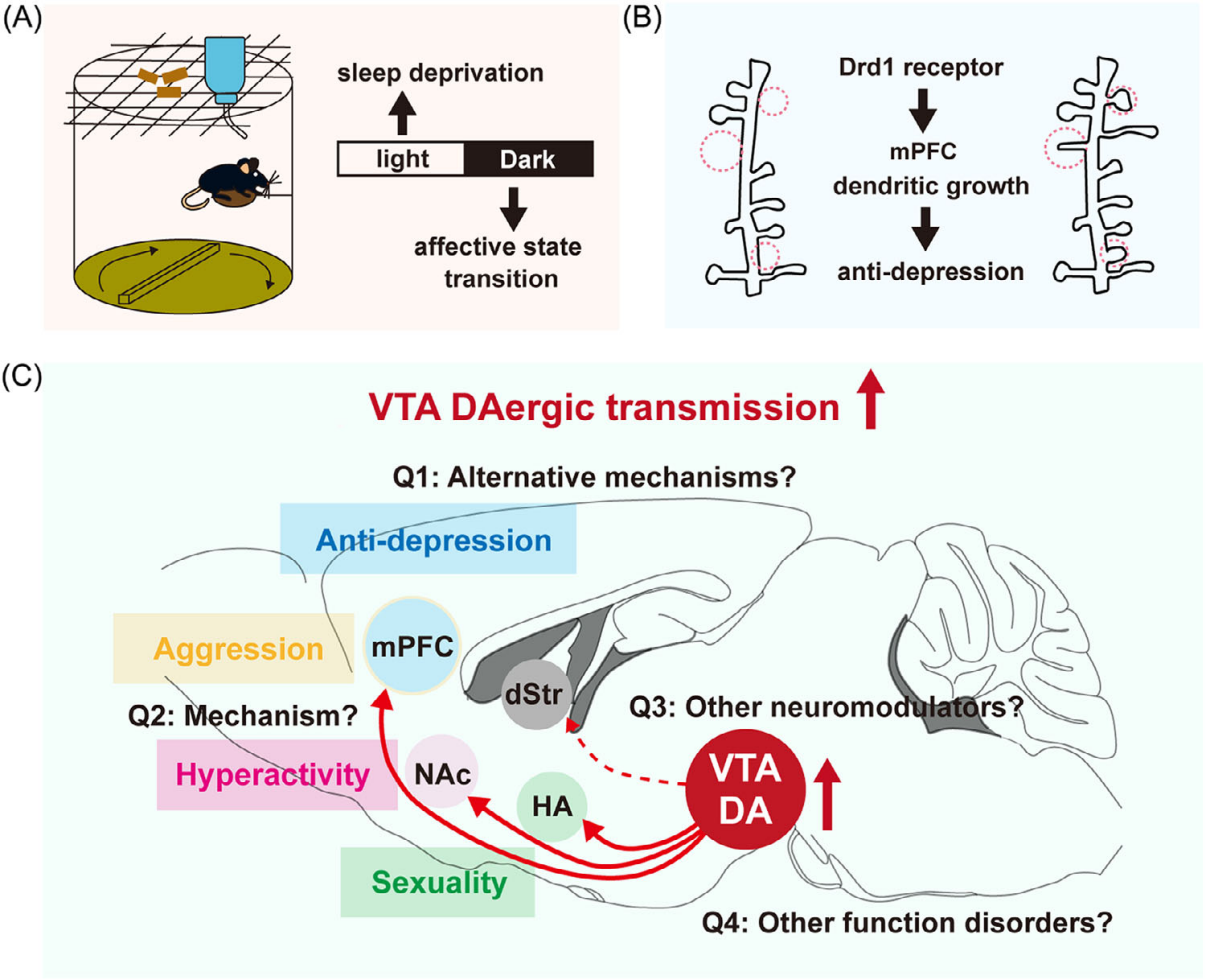
Distinct VTA DAergic subsystems mediate diverse affective alterations induced by SD, raising several directions for further investigation. (A) Scheme of experiment for automatic sleep deprivation in mice. (B, C) Acute sleep deprivation elicits a pronounced augmentation in VTA‐mPFC, VTA‐NAc, and VTA‐HA DAergic transmissions, thereby instigating an escalation in aggression, hyperlocomotion, sexuality, and antidepressant effects. Drd1 receptor‐dependent alteration of neuronal plasticity in the mPFC is required for the antidepressant effects of acute SD. However, several questions need to be further addressed: (1) Is there any alternative mechanism for the immediate antidepressant effect of SD? (2) What is the mechanism underlying mania‐like alterations induced by SD? (3) What is the role of other neuromodulators in SD‐induced affective state transition? (4) What are the effects of SD on other physiological functions? Abbreviations: DA, dopamine; dStr, dorsal striatum; HA, hypothalamus; mPFC, media prefrontal cortex; NAc, nucleus accumbens; SD, sleep deprivation; VTA, ventral tegmental area.

The DAergic nervous system, as a vital component of the ascending reticular system, plays a crucial role in essential physiological functions such as sleep‐wake regulation, emotional modulation, and motor coordination.^3^ Moreover, alterations in the function of distinct DAergic neural circuits can contribute to various affective disorders including depression and mania.^4^ However, the precise function and underlying circuit mechanism of the DAergic system implicated in the emotional state transition induced by sleep rhythm disturbance remain to be elucidated. Using calcium fiber photometry, Wu et al. observed a remarkable increase in the calcium activity of DAergic neurons in the ventral tegmental area (VTA) during acute SD.^1^ Chemogenetic inhibition of VTA DAergic neurons during SD not only reversed the SD‐induced hyperactivity and aggressive behavior but also reduced the attenuation in depressive behaviors while having no effect on the sexual behavior. These findings suggest that acute SD induces increased activity in VTA DAergic neurons, leading to an affective state transition in mice.

Increasing evidence indicates that the DAergic systems contribute to various physiological behaviors via distinct downstream circuits. To investigate alterations of DAergic transmission in different downstream brain regions, Wu et al. used dopamine sensors to monitor dopamine release in the nucleus accumbens (NAc), the medial prefrontal cortex (mPFC), the hypothalamus (HA), and the dorsal striatum (dStr).^1^ During SD, a significant increase in dopamine content was observed within the NAc, the mPFC, and the HA, while no change was detected in the dStr. They further applied circuit‐specific modulation to evaluate their functions. Selectively inhibition of the VTA‐NAc DAergic circuit reversed SD‐induced increases in locomotion, and selective inhibition of the VTA‐mPFC DAergic circuit reversed increases in aggressive behavior and antidepressant effects. Intriguingly, selective inhibition of the VTA‐HA DAergic circuit counteracted the elevation in sexual behavior, whereas selective inhibition of the VTA‐dStr DAergic circuit did not exert any influence on these aforementioned behaviors. These findings demonstrate that the alterations in various behaviors of SD mice are mediated by distinct DAergic subsystems that project to different downstream targets. Furthermore, except for dopamine, neuromodulators including histamine and serotonin also play a pivotal role in waking‐promoting systems and are intricately involved in the regulation of emotion. Therefore, investigating their contribution to affective state transitions induced by SD is equally imperative.

Alterations in neuroplasticity within the mPFC are closely associated with depression. Wu et al. previously demonstrated that ketamine enhances dopamine transmission in the mPFC and induces hyperplasia of dendritic spines on pyramidal neurons via dopamine receptor D1 (Drd1) receptors, thereby exerting rapid antidepressant effects.^5^ They wonder if SD‐induced VTA‐mPFC DAergic hyperactivity also results in rapid antidepressant effects through similar mechanisms. By using Thy1‐EGFP mice, they found a significant increase in the density of dendritic spines on deep pyramidal neurons in the mPFC 24 h following SD, they also detected an immediate enhancement in the probability of glutamate‐induced dendritic spines regeneration after acute SD. Besides, conditional knockout of the Drd1 receptor in the mPFC reversed the alteration of dendritic spine regeneration. These results suggest that acute SD induces a Drd1 receptor‐dependent alteration of neuronal plasticity in the mPFC. In the end, Wu et al. used genetically encoded photoactivatable Rac1 (PaRac1) to selectively eliminate recently activated synapses in the mPFC and found that activating PaRac1 24 h after SD significantly reversed the antidepressant effects. These findings suggest that Drd1 receptor‐dependent dendritic spine plasticity is required for the antidepressant effects of acute SD. Overall, The VTA‐mPFC DAergic circuit mediates an antidepressant effect through Drd1 receptor‐dependent regeneration of dendritic spines. However, inhibiting neonatal dendritic spines in the mPFC did not reverse the immediate antidepressant effect after SD, suggesting the existence of alternative mechanisms.

In conclusion, this study demonstrates that an increase in distinct DAergic transmissions contributes to diverse behavior alterations during SD‐associated affective state transitions (Figure 1B,C). In addition to affecting emotional states, SD can also impact other crucial physiological functions including cognition and memory. This automatic SD mouse model can be further used to explore various other function alterations associated with SD.

## AUTHOR CONTRIBUTIONS

Ju Lan and Heming Cheng wrote the manuscript and prepared the figure. Zhong Chen provided valuable discussion and modified the manuscript. All authors have read and approved the final manuscript.

## CONFLICT OF INTEREST STATEMENT

The authors declare no conflict of interest.

## ETHICS STATEMENT

Not applicable.

## Data Availability

Not applicable.
